# Research and lobbying conflicting on the issue of a front-of-pack nutrition labelling in France

**DOI:** 10.1186/s13690-016-0162-8

**Published:** 2016-11-28

**Authors:** Chantal Julia, Serge Hercberg

**Affiliations:** 1 Université Paris 13, Equipe de Recherche en Epidémiologie Nutritionnelle (EREN), Centre d’Epidémiologie et Biostatistiques Sorbonne Paris Cité (CRESS), Inserm U1153, Inra U1125, Cnam, COMUE Sorbonne-Paris-Cité, F-93017 Bobigny, France; 2 Département de Santé Publique, Hôpital Avicenne (AP-HP), F-93017 Bobigny, France

**Keywords:** Front-of-pack nutrition labels, Lobbies, Agro-industry, Public health policies

## Abstract

Front-of-pack nutrition labelling has been highlighted as a promising strategy to help consumers making healthier food choices at the point of purchase. In France, a simplified front-of-pack nutrition labelling system was proposed in 2014, the 5-Colour Nutrition Label (5-CNL). It is supported by studies evaluating the various dimensions of the validation of both its underlying classification algorithm and its format. Opposed by agro-industry and retailers, multiples lobbying strategies have been deployed to stop or at least delay the implementation of the 5-CNL. Various alternative nutrition labels were proposed, and a full-scale trial was successfully argued for. This paper retraces the various steps of the opposition between public health and agro-industry lobbies on the topic of front-of-pack nutrition labelling in France.

## The case for front-of-pack nutrition labelling

The burden of non-communicable diseases is growing worldwide, partly fuelled by the growing prevalence of overweight and obesity [1, 2]. Diet has long been identified as a modifiable risk factor for multiple non-communicable diseases, including obesity, and therefore appears as a key lever to prevent future deterioration of the health status of the population [1, 3–6]. Most western countries have put up state-level programs on nutrition, addressing both individual and environmental determinants of dietary behaviour [7, 8]. France launched in 2001 the National Nutrition and Health program (Programme National Nutrition Santé, PNNS) [9]. It aims at improving the health of the population by acting on nutrition, through multifaceted and multilevel interventions, including regulations, education and multimedia information campaigns or local initiatives of health promotion. However, though in existence for more than fifteen years now, its contribution to the improvement of nutritional status and prevention of overweight and obesity appears limited [9]. Though the prevalence of obesity in children appears to have stabilized [10], it is still growing in adults, and social inequalities in overweight and obesity prevalence have risen.

Novel strategies have been highlighted as promising by various learned societies to incorporate in state-level nutrition programs, and those include advertising regulations, taxation of unhealthy foods and beverages, or front-of-pack nutrition labelling [11, 12]. A report to the French Minister of Health by the president of the PNNS in January 2014 highlighted all three strategies as novel pathways to promote a healthier dietary environment in France [13]. In particular, a simplified front-of-pack nutrition labelling system was proposed, the 5-Colour Nutrition Label (5-CNL).

## The French proposal

**Fig. 1:**
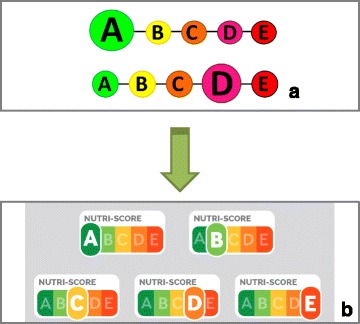
5-Colour Nutrition Label as presented in the report from Pr. Hercberg en January 2014 (**a**) and its evolution following a study by INPES (Institut National de Prévention et d’Education pour la Santé, National Health Prevention and Education Institute) in October 2015 (**b**)

Based on the British Food Standards Agency nutrient profiling system (FSA score), currently in use for the regulation of advertising to children in the United Kingdom [14, 15], the 5-CNL classifies foods and beverages according to five classes of nutritional quality, on a colour scale ranging from Green (with the grade A) to red (with the grade E). The scale is presented in its entirety, so that the consumer can easily identify the level of nutritional quality of the product compared to the whole scale (see Fig. 1).

The concept of a front-of-pack nutritional label was welcomed by the Minister, and included in the draft to the 2016 Health law in April 2015 [16]. French independent sanitary agencies were notified to investigate the feasibility of the use of the FSA score for a five-category nutrition label, and the relevance of the proposed format of the label in the light of the current literature on the subject and the existing formats being in use in the world.

Parallel to this political agenda, epidemiologic and experimental studies were conducted by independent research units on this label investigating both the validity of the nutrient profiling system underlying the label and the perception, objective understanding and use of the label itself.

## Literature supporting the label

Studies on the validity of the nutrient profiling system analysed the consistency between the classification by the FSA score – used as a basis for the classification of foods and 5-CNL colour attribution – and French nutritional recommendations, using three dietary composition databases [17–19]. They concluded that the FSA score was consistent with dietary recommendations, provided some amendments to the original score for cheese, beverages or added fats were included. Moreover, they showed that an individual dietary index based on the FSA score of the foods consumed – the FSA-NPS DI – was able to characterize the nutritional quality of the individual’s diet [20, 21]. Finally, the FSA-NPS DI was found to be associated with cardiovascular disease, cancer, metabolic syndrome and obesity in men, supporting the use of the FSA score for public health policies [22–25].

Moreover, studies comparing the 5-CNL to other formats currently in use worldwide (namely the British *Multiple Traffic Light*, the *Guidelines Dietary Amounts* and a ‘Check’ label, close to the Ducth *Choices* label) found that the 5-CNL was considered easy to identify and understand, that it was helpful to consumers to classify foods according to their nutritional quality, and that it was associated to a higher quality of the items in the purchasing cart in a randomized trial set in an online experimental supermarket [26–28]. These latter results were confirmed using a physical experimental supermarket, where the presence of the 5-CNL label, combined with a communication leaflet was associated with a higher nutritional quality of the sweet biscuits purchased [29]. Finally, in another experimental design, comparing the impact of nutrition labelling on a pre-elaborated shopping basket, the positive impact of the 5-CNL on the nutritional quality of purchases was again confirmed [30].

The report commissioned by the government from the French Agency for Food, Environmental and Occupational Health & Safety (Agence nationale de sécurité sanitaire de l’alimentation, de l’environnement et du travail, ANSES) confirmed in April 2015 that the FSA score could be easily computed using data already present on back-of-pack nutritional labelling (it could be calculated on more than 12,000 products as sold in France), and that it could be used for labelling purposes [31]. Some limitations to the algorithm were identified for some food groups, for which the original score did not allow to discriminate foods, such as beverages or added fats [31]. The report from the High Council of Public Health (Haut Conseil de la Santé Publique, HCSP) completed the report from the ANSES by adding modifications to the original FSA algorithm for the food groups (beverages, cheese and added fats) that had been previously identified in order to ensure a high consistency between the classification of foods according to the score and nutritional recommendations [32]. Moreover, it set the various cut-offs for the classes of the 5-CNL, and finally, after a review of the various labels currently proposed in the world, it considered that the 5-CNL was the only relevant one in the French environment [32].

## The reaction of economic operators

The reaction of economic operators to the proposal was a firm opposition to the 5-CNL. Immediately after the Minister announced her intention of including the label in the Health law, the confederation of French agri-industry (Association Nationale des Industries Alimentaires, ANIA) opposed it, qualifying it of ‘a discriminatory measure based on a simplistic and functional approach to foods’ [33]. It was also deemed to be ‘a restriction on exports’ and ‘a threat to the economic and social dynamics of SMEs and territories’ [34].

The ANIA went a step further arguing that a front-of-pack label should take into account ‘portion size, eating occasion, frequency of consumption and the possible association of foods’ [33]. Though no scientific study ever reported a front-of-pack label to induce guilt in consumers and that a simple format by no means implies a simplistic one, it has to be underlined that all the above mentioned characteristic of a given food to be taken into account in a single front-of-pack label is simply unrealistic. The number of combinations between associations of foods, portion size and eating occasion for a given food are limitless, and depend so much on individual characteristics that the inclusion of them in any system would entail major simplifications and shortcomings.

After this direct opposition, agro-industry and retailers lobbies worked to slow down the potential implementation of the label at national level, through various strategies.

First, they developed and promoted their own labelling systems, often without any scientific validation. One of the major retailers in France, Carrefour, announced its own label as soon as September 25, 2014 [35]. This label was later endorsed by multiple retailers, and renamed under ‘SENS’. Though the format of the label, developed internally by marketing teams, varied only slightly in time, its underlying algorithm, developed by a research team, was disclosed only in December 2015, more than a year later [36]. Other manufacturers openly supported other formats, such as the British *Traffic Light* system, a modified version of the *Reference Intakes* or even the Australian *Health Star Rating system* during the year 2015. Noteworthy, all these systems had been reviewed by the High Council of Public Health, and discarded compared to the 5-CNL in its report of August 2015 [32]. The SENS format had also been openly criticized by scientists and consumer associations as soon as October 2014 [37]. Indeed, the SENS format, including only four categories – with only one present on the front-of-pack, the entire scale not being directly accessible – introduces directly on the label a notion of frequency of consumption (“Very often”, “Often”, “Regularly in small portions or moderately” and “occasionally”), which have no scientific validation whatsoever [32]. Moreover, the colours of the label do not refer to any known perceptual graduation, as they include green, blue, orange and violet. As to the others, nutrient-specific labels were considered by the High Council of Public Health as less easily understandable than simple ones, in particular to socially disadvantaged groups, and the *Health Star Rating System* was discarded because the underlying algorithm required nutrients that were not easily accessible [32].

However, such advisory information was considered insufficient, and the ANSES was notified to compare the algorithms of the 5-CNL and the SENS in early 2016. The report showed that the results of the classification using the SENS algorithm did not substantially differ from the 5-CNL classification. However, it required unavailable nutrient information for its computation, and the ANSES was therefore not able to calculate it on the 12,000 products it had included in the 5-CNL report, but only 1.100 generic foods [38].

In a second strategy to slow down the adoption of the measure, agro-industries lobbied to obtain a ‘full-scale test in real conditions’ to test the various formats that had been put forward, including the 5-CNL and the alternative labels from industry and retailers. Amendments directly produced by lobbies introducing the principle of an experimental study beforehand were proposed during parliamentary sessions, and rejected by the government. The Health law was adopted in December 2015, and therefore did not mention any experimentation before the implementation of the label.

However, lobbies obtained that a ‘full-scale test in real conditions’ was to be performed, testing four of the options put forward during the discussions: the 5-CNL, SENS, the *Multiple Traffic Lights* and a modified version of the *Reference Intakes*. Moreover, they obtained that the piloting of the project would be entrusted to a private party, and not to independent or academic structures. The Fond Français pour l’Alimentation et la Santé (FFAS), which was selected, is entirely funded by agro-industry and retailers. The experimental study’s design and protocol was entrusted to a scientific committee, and was overseen by a piloting committee including officials from various ministries and directors of independent research agencies. The piloting committee also included representatives from agro-industry and retailers. However, three members of the scientific committee resigned to protest against the fact that some members of the committee were funded by industry, and that their corrections and contributions to the protocol were not taken into account. Finally, the president of Inserm, the national health research agency also resigned from the piloting committee, considering the protocol was not scientifically sound. Despite these reservations from scientists, which were disclosed in the press, the experimentation is to be carried forward.

Finally, lobbies attempted to stop ongoing public research on the 5-CNL, by applying directly to the Minister of Agriculture, arguing that conducting such research could bias the results of the experimentation. In particular, they argued that the mediatisation of the results of such a study would act as a marketing campaign promoting the 5-CNL [39].

These strategies led to a considerable delay in the implementation of the law. The implementing order for the label will not be ready before the next French presidential election in 2017, and could therefore be entirely stopped by these manoeuvres for several years.

The media recently uncovered these various strategies, prompting a surge in the public opinion against such lobbying. A petition promoting the direct adoption of the 5-CNL on the Change.org platform rapidly reached near 200,000 signatures following an investigation from French journalists. However, no response to this initiative was received from public officials, except that the experimentation should be carried forward.

## Conclusion

These delaying tactics once again demonstrate the potential harm to citizens’ information when public health proposals directly conflict with the economic growth of agro-industry.

## Abbreviations

5-CNL: 5-Colour Nutrition Label
ANIA: Confederation of French agri-industry, Association Nationale des Industries Alimentaires
ANSES: French Agency for Food, Environmental and Occupational Health & Safety, Agence nationale de sécurité sanitaire de 1’alimentation, de 1’environnement et du travail
FFAS: Fond Français pour 1’Alimentation et la Santé
FSA score: Food Standards Agency nutrient profiling system
FSA-NPS DI: Food Standards Agency Nutrient Profiling system Dietary Index
HCSP: High Council of Public Health, Haut Conseil de la Santé Publique
PNNS: French National Nutrition and Health Program, Programme National Nutrition Santé
